# Giant filarial retroperitoneal cyst: a diagnostic dilemma

**DOI:** 10.1186/s41182-019-0164-7

**Published:** 2019-06-13

**Authors:** Pawan Lal, Lovenish Bains, Gaurish Sawant, Rahul Saini, Shramana Mandal

**Affiliations:** 1 Department of Surgery, Maulana Azad Medical College, New Delhi, India; 2 Department of Pathology, Maulana Azad Medical College, New Delhi, India

**Keywords:** Filariasis, Retroperitoneal, Computed tomography, Cyst, Lymphatic

## Abstract

**Background:**

Filarial infections are common in most tropical and subtropical regions of the world. Lymphatic filariasis is caused by either *Wuchereria bancrofti*, *Brugia malayi*, or *Brugia timori.* Extralymphatic filariasis presenting as a primary retroperitoneal mass is very rare despite filariasis being endemic in many regions of India. On review of literature, only a few isolated case reports have been described.

**Case presentation:**

We report a case of a huge retroperitoneal cystic mass in a 46-year-old patient who presented with a long-standing, painless progressive abdominal swelling. On examination, there was a large, non-tender, firm swelling of size around 20 × 15 cm occupying the left upper and lower quadrant. The computed tomography of the abdomen was suggestive of thin-walled hypodense cyst of size 25.7 × 15 × 14.3 cm. Laboratory investigations and cyst aspirate were inconclusive for a definite diagnosis. On exploration, a 3-kg cystic mass was removed. The diagnosis of filarial origin was confirmed by the demonstration of microfilaria in the cyst wall and immunochromatographic test (ICT) which was positive.

**Conclusion:**

Retroperitoneal lymphatic cyst of filarial origin is very unusual and requires a high index of suspicion if the patient is an inhabitant of an endemic area. The clinical dilemma cannot be resolved with imaging modalities alone, unless a disease-specific manifestation is there. The retroperitoneal cysts often pose a challenge in their diagnosis and management. Small cysts might respond to medical management, whereas large symptomatic cysts will require excision for the final diagnosis and treatment.

## Introduction

Retroperitoneal cysts are uncommon masses whereas primary filarial retroperitoneal cysts are rare [1]. The reported incidence of filarial retroperitoneal cyst in hospitalized patients is 1 in 105,000 patients [2]. The clinical manifestations of lymphatic filariasis are due to occlusion of the lymphatic channels, thereby causing lymphangiectasia. The extralymphatic filarial disease is multifactorial in its pathogenesis and may be not caused by the adult worm per se. It is postulated to be caused by microfilariae or by diffusible products from yet undefined parasitic stages. Primary retroperitoneal parasitic cysts are uncommon [3] and majority of them are hydatid cysts. Retroperitoneal filarial cysts are very rare even in endemic countries like India. Extensive scrutiny of indexed literature like “PubMed” including old Indian journal archives with keyword “retroperitoneal filarial cyst” resulted in 10 isolated cases till date all of which happened to be from India.

### Case report

**Fig. 1:**
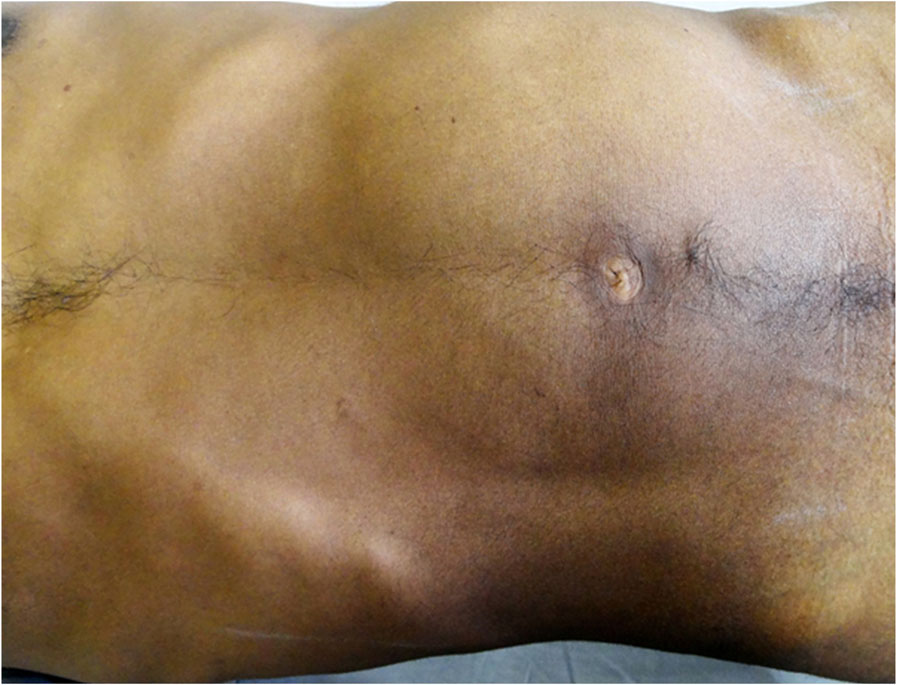
Swelling occupying whole of the left side of the abdomen

A 46-year-old male patient, a native of district Gorakhpur, Uttar Pradesh state, India, in October 2017 presented to us with complaints of a painless progressive swelling in the left side of the abdomen with abdominal distension for the last 8 years. The swelling was not associated with any symptom therefore the patient did not seek any medical attention for the same; however, for the last 2 years, the swelling has increased considerably as per the patient and causes dragging discomfort. It was not associated with any bladder or bowel complaints or any other systemic symptoms like fever, weight loss, or loss of appetite. Apart from dragging discomfort, there was no history of anorexia, paroxysmal hypertension, tachycardia, headache, perspiration, or palpitations. The socioeconomic status was lower middle (class III) as per modified Kuppuswamy scale [4], and the patient was a farmer by profession. On abdominal examination, there was a large, firm swelling of size around 20 × 15 cm occupying the left upper and lower quadrant (Fig. 1). It was non-tender and dull on percussion. The scrotum and testis were normal and there was no pedal edema or lymphadenopathy. A provisional clinical diagnosis of pseudo-pancreatic cyst was made.

**Fig. 2:**
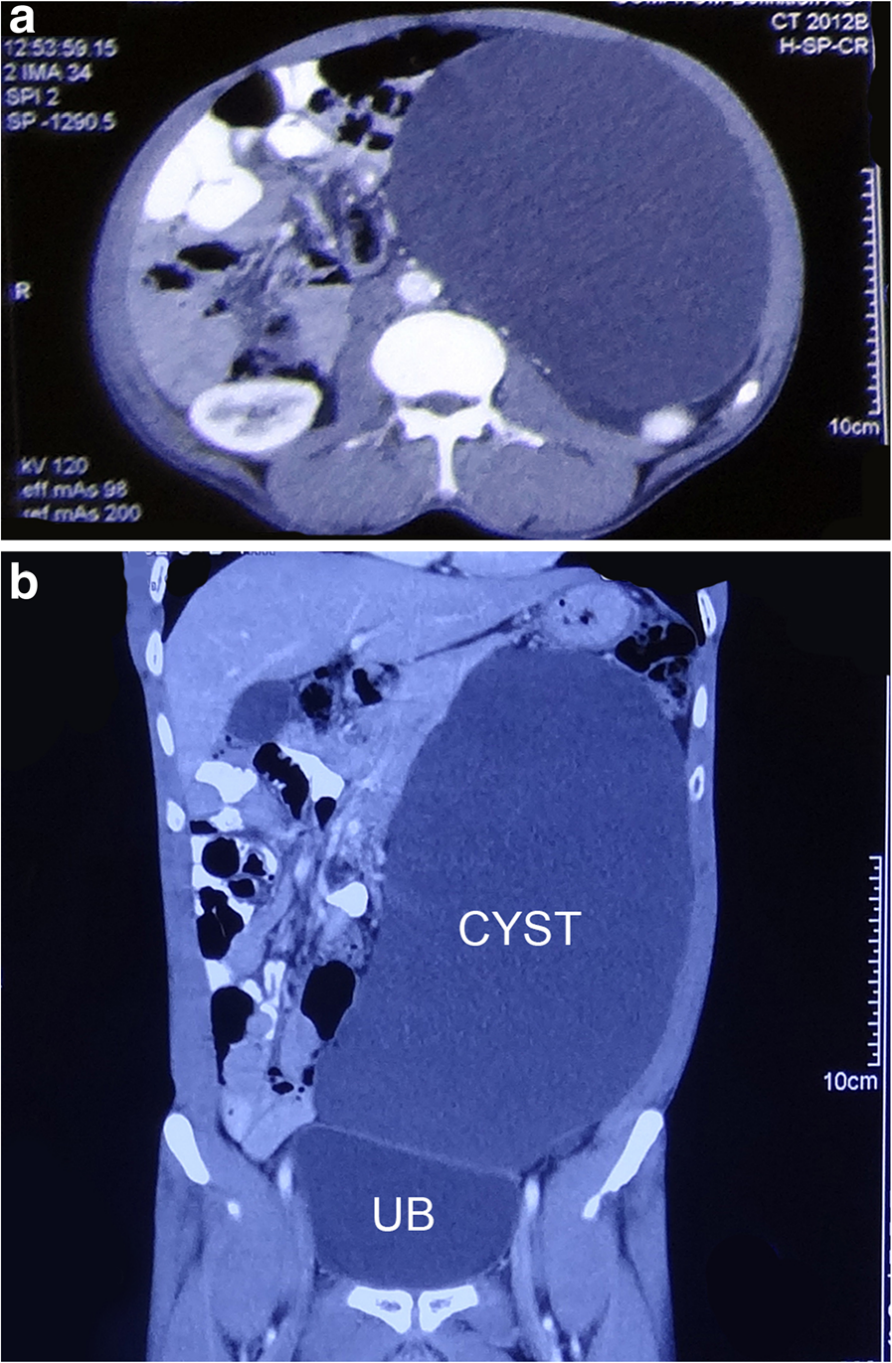
**a** Hypodense cystic mass displacing the bowel loops, abdominal aorta, and superior mesenteric vessels to the right side. **b** Coronal section of CECT showing a cyst occupying almost whole of the abdomen. UB urinary bladder

Blood and laboratory investigations were within normal limits. Abdominal contrast-enhanced computed tomography was suggestive of a thin-walled hypodense cystic mass of size 25.7 × 15 × 14.3 cm in the left side of the abdomen extending from the lesser sac till the left iliac fossa. The lesion was compressing the body and tail of the pancreas. It was also displacing the head of the pancreas, stomach, first and second parts of the duodenum, small bowel loops, abdominal aorta, and superior mesenteric vessels to the right side. It was compressing the left ureter causing mild hydro-ureteronephrosis. The head of the pancreas was mildly bulky but there was no focal lesion and no evidence of free fluid or lymph nodes in the abdomen suggesting a possible diagnosis of cystic pancreatic lymphangioma (Fig. 2a, b). Twenty-four-hour urinary metanephrines were also done to rule out a cystic retroperitoneal paraganglioma and the test was negative. The diagnostic aspiration of cyst fluid (done in December 2017) revealed a total cell count of 85 cells/mm^3^ with 60% lymphocytes and 40% neutrophils whereas glucose was 45 mg/dl, protein 4.5 g/L, and amylase 24 U/L. The culture of cyst fluid was sterile and malignant cytology was negative.

**Fig. 3:**
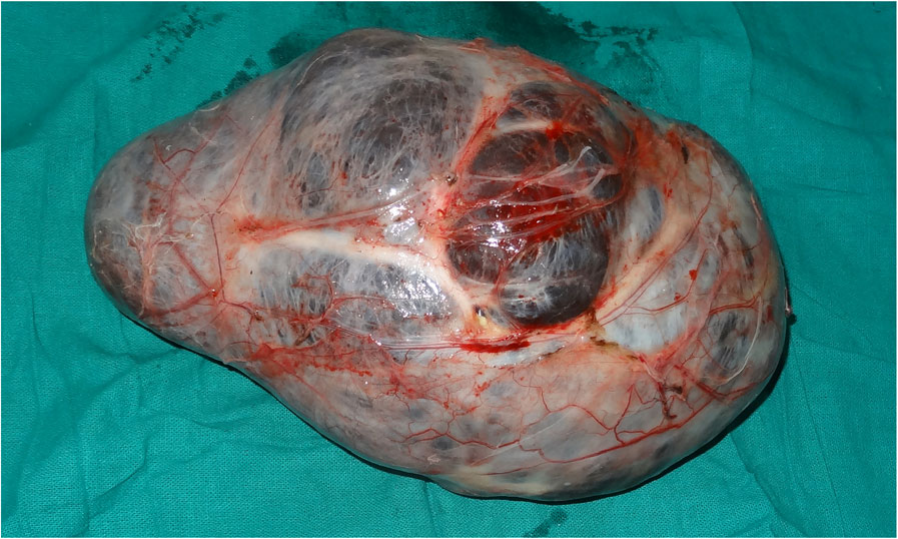
Excised specimen retroperitoneal cystic mass of size 25 × 15 × 15 cm

The patient posed to us a diagnostic dilemma and we were unable to reach a definite diagnosis even after extensive investigations. The patient was planned for exploration with a probable diagnosis of pancreatic pseudocyst or lymphangioma. The patient was operated on February 2018, on exploration, and there was a cystic mass of size 25 × 15 × 15 cm (Fig. 3), which was present in the retroperitoneum pushing the small and large bowel loops anteriorly and to the right. The ureter and gonadal vessels were compressed posteriorly by the mass. Cranially, it was pushing the stomach and pancreas to the right, but there was no obvious connection with the pancreas. There were no obvious dilated lymphatics in the retroperitoneum.

**Fig. 4:**
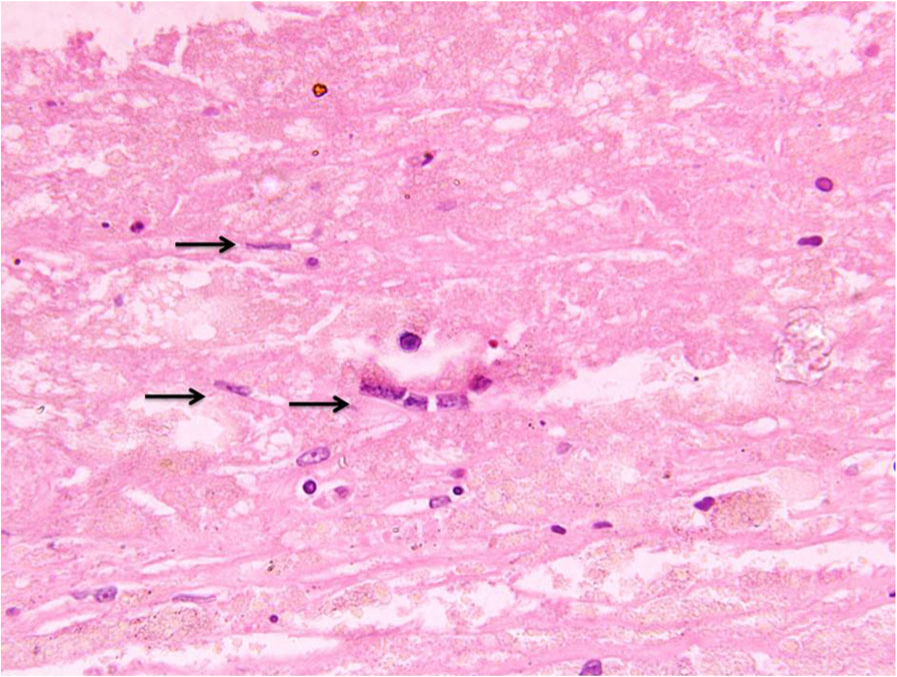
H&E stain (400 ×) showing microfilarial fragment in a background of fibro-collagenous

Histopathology of the cyst revealed a unilocular cyst with walls composed of fibro-collagenous tissue lined by flattened epithelium and focal areas of calcification along with a few fragments of microfilaria (Fig. 4). The features were suggestive of a lymphatic cyst of filarial origin. The postoperative recovery of the patient was uneventful and was given diethylcarbamazine therapy (100 mg t.i.d. for 3 weeks). Further, filariasis immunochromatographic test (ICT) by Alere™ BinaxNOW® Filariasis kit for *Wucheria bancrofti* was positive. Ultrasound of the scrotum, groin, and lower extremity was reviewed again for possible adult worm and, however, was negative. The patient was doing fine up to 8 months of follow-up.

## Discussion

Filariasis is a parasitic zoonotic endemic infection seen in the tropical and subtropical regions of the world. Approximately 120 million people are infected all over the globe whereas about 856 million people in 52 countries worldwide remain threatened by lymphatic filariasis and require preventive chemotherapy to stop the spread of this parasitic infection^.^[5]. Lymphatic filariasis has been identified by the World Health Organization (WHO) as a major cause of disability worldwide, with an estimated 40 million individuals affected by the disfiguring features of the disease [6]. Fifty-seven percent of the total population requiring preventative chemotherapy live in the South East Asia Region (nine countries) and 37% live in the African Region (35 countries) [7]. As per WHO estimate, 449.3 million people required MDA in 2016 in southeast Asia, out of which, about 75% reside in India [8].

Worldwide, 90% of infections are caused by *Wuchereria bancrofti* and the remainder by *Brugia* spp. whereas in India, 99.4% of the cases are caused by the *Wuchereria bancrofti* and other species *Brugia malayi* responsible for 0.6%. It commonly presents with lymphatic dysfunction in the form of lymphocele, hydrocele, chyluria, or groin lymphadenovarix along with severe disability and social stigma. The clinical manifestations of extralymphatic disease caused by filariasis are multiple and range from symptoms due to tropical pulmonary eosinophilia to skin rashes, hematuria, proteinuria, granulomata, subcutaneous nodules, splenomegaly, and rarely arthritis.

Cystic retroperitoneal mass may be due to many causes including benign lesions such as lymphocele, urinoma, mesothelial cyst, lymphangioma, traumatic cyst, and parasitic cyst, as well as malignant lesions such as mucinous cystadenocarcinoma, pseudomyxoma retroperitonei, parachordoma, and cystic teratoma [9]. In this patient, the possibilities of lymphangioma and pancreatic pseudocyst were kept as differential diagnosis.

CT is the investigation of choice as it may provide important information regarding lesion location, size, and shape; the presence and thickness of a wall; the presence of septa, calcifications, or fat; and involvement of adjacent structures [10].

Despite extensive imaging evaluation, the diagnosis very often can only be obtained on some form of histopathology such as aspiration of fluid and cytology or excision and biopsy. Often, primary retroperitoneal parasitic cysts are hydatid cysts [4]. Primary retroperitoneal cyst of filarial origin is an extremely uncommon entity, though a variety of extralymphatic manifestations of filariasis have been described in the literature [2, 3, 10]. Whenever there is a strong doubt about such cysts, especially in patients from endemic regions, filarial antigen tests or DNA assays may provide some supporting information. Filariasis causing retroperitoneal cyst is rare even in regions where filariasis is endemic. Only 10 prior cases of retroperitoneal filarial cyst were found after extensive search of literature (Table 1). All cases happened in male patients with a mean age of 36 years (range 21–52).

**Table 1:** Retroperitoneal filarial cyst as mentioned by various authors

Year	Author	Cyst size	Age (in years)	Sex	Site (in retroperitoneum)	Region, if specified (country, India)
1961	Pillay [11]	25 × 10 cm	26	Male	Right iliac fossa and pelvis	
1981	Mehta et al. [1]	8 × 10 cm	22	Male	Filarial abscess	
1987	Chittipantulu et al. [12]			Male		South India
1989	Gupta et al. [13]	20 × 15 cm	40	Male	Displacing stomach, transverse and descending colon	
2000	Giri et al. [14]	5.5 cm × 5.3 cm	38	Male	Anterior to left psoas muscle and common iliac vessels partially compressing the left ureter causing left-sided hydroureter and hydronephrosis	Darjeeling, Bengal
2011	Kapoor [9]		42	Male	Right hypochondrium to the infraumbilical region	North India
2015	Srivastava et al. [15]	Large cyst, exact size not mentioned	21	Male	Retroperitoneum	Rural North India
2015	Ganesan et al. [16]	20 × 14 × 23 cm	35	Male	Behind the right colon, mesocolon	Rural north India
2016	Bakde et al. [17]	21 × 10 cm	38	Male	Right hypochondrium to the pelvic region	Urban Maharashtra
2018	Diwakar et al. [18]	9 × 8 cm	52	Male	Right lumbar region	Rural north India
2019 (current case)	Lal P, Bains L et al.	25 × 15 × 15 cm	46	Male	Left side of the abdomen extending from the lesser sac till the left iliac fossa	District Gorakhpur, Uttar Pradesh

The exact pathogenesis of the extralymphatic manifestation remains speculative. The dilatation of retroperitoneal lymphatic channels due to obstruction and their rupture and the presence of ectopic lymphatic tissue have been proposed as the possible etiologies [3].

The peculiar presentation of this patient of a long-standing retroperitoneal cyst, without other common manifestations of filariasis like hydrocele and lymphedema, was adding diagnostic dilemma. It was decided to perform surgery as the patient had large retroperitoneal cyst with diagnostic uncertainty even after contrast-enhanced computed tomography and cyst fluid analysis. The final diagnosis was reached, based on a microscopic demonstration of fragments of microfilariae in the cyst wall and positive filarial antigen ICT test. Small retroperitoneal lesions may resolve with antifilarial therapy, but most cases including our case require surgical removal because of their large size [10, 14, 15].

## Conclusion

Retroperitoneal lymphatic cyst of filarial origin is very unusual and requires a high index of suspicion if the patient is an inhabitant of an endemic area. The clinical dilemma cannot be resolved with imaging modalities alone, unless a disease-specific manifestation is there. The retroperitoneal cysts often pose a challenge in their diagnosis and management. Small cysts might respond to medical management, whereas large symptomatic cysts will require excision for the final diagnosis and treatment.

## Data Availability

Not available.

## Abbreviations

ICT: Immunochromatographic test
MDA: Mass drug administration
WHO: World Health Organization
